# The Peculiar Characteristics of Fish Type I Interferons

**DOI:** 10.3390/v8110298

**Published:** 2016-11-02

**Authors:** Pierre Boudinot, Christelle Langevin, Christopher J. Secombes, Jean-Pierre Levraud

**Affiliations:** 1 Virologie et Immunologie Moléculaires, INRA, Université Paris-Saclay, 78350 Jouy-en-Josas, France; Christelle.Langevin@jouy.inra.fr; 2 Scottish Fish Immunology Research Centre, School of Biological Sciences, University of Aberdeen, Aberdeen AB24 2TZ, UK; c.secombes@abdn.ac.uk; 3 Macrophages et Développement de l’Immunité, Institut Pasteur, 75015 Paris, France; 4 Centre National de la Recherche Scientifique, Unité Mixte de Recherche 3738, 75015 Paris, France

**Keywords:** type I IFN, fish innate immunity, evolution

## Abstract

Antiviral type I interferons (IFNs) have been discovered in fish. Genomic studies revealed their considerable number in many species; some genes encode secreted and non-secreted isoforms. Based on cysteine motifs, fish type I IFNs fall in two subgroups, which use two different receptors. Mammalian type I IFN genes are intronless while type III have introns; in fish, all have introns, but structurally, both subgroups belong to type I. Type I IFNs likely appeared early in vertebrates as intron containing genes, and evolved in parallel in tetrapods and fishes. The diversity of their repertoires in fish and mammals is likely a convergent feature, selected as a response to the variety of viral strategies. Several alternative nomenclatures have been established for different taxonomic fish groups, calling for a unified system. The specific functions of each type I gene remains poorly understood, as well as their interactions in antiviral responses. However, distinct induction pathways, kinetics of response, and tissue specificity indicate that fish type I likely are highly specialized, especially in groups where they are numerous such as salmonids or cyprinids. Unravelling their functional integration constitutes the next challenge to understand how these cytokines evolved to orchestrate antiviral innate immunity in vertebrates.

## 1. Introduction

Interferons (IFNs) are the hallmark of vertebrate innate immunity to viruses. IFNs belong to the class II helical cytokine family, and can be classified into three families (type I IFNs, which include IFNα and -β, type II or γ IFN, and type III or λ IFNs) based on their structural and functional properties, and on their receptors. Both type I IFNs and type III IFNs are strongly induced by viral infections and play a major role in the early innate response against viruses.

In contrast, type II IFNs are immunoregulatory cytokines with key roles in both innate and adaptive immunity, notably to intracellular bacteria, and are mainly produced by activated natural killer (NK) and T helper 1 cells. Type II IFNs are also present in fish [1], but they will not be discussed in this review. Following the discovery of the induction of type I IFNs activity by influenza virus [2], human IFNα and -β genes have been cloned [3,4], and an impressive diversity of type I IFN genes was discovered. Anti-proliferative functions were also demonstrated, and type I IFNs have been used to fight different cancers as well as viral infections [5].

Mammalian type I IFNs bind the IFN-alpha/beta receptor subunit 1/2 (IFNAR1/IFNAR2) complex, and signal through the Jak/STAT pathway involving Jak kinases Jak1 and Tyk2 and STAT transcription factors (STAT-1 and -2, principally). Cellular response to IFN then consists of the up-regulation of a large number of genes, named ISG (for “IFN stimulated genes”); some ISG have a direct antiviral activity, while others are modulators of the signalling pathways upstream or downstream of IFN itself. A number of ISG can also be induced by viral infections in an IFN-independent manner, for example via interferon regulatory factor 1 (IRF1). The functional complexity of type I IFN has been recently dissected in several large-scale functional screens [6,7].

The production of molecules with antiviral IFN-like activity by fish cells was described in the 1970s [8], but fish type I IFN genes were not cloned and identified until 2003 [9,10,11], due to failure of approaches based on polymerase chain reaction (PCR) with primers derived from mammalian IFN sequences. Gene identification in expressed sequence tag (EST) and genomic libraries opened the way to the characterization of the fish IFN system, including IFN receptors, key factors of the signalling pathways leading to IFN and ISG induction, and ISG repertoires. These studies revealed an astonishing diversity of fish type I IFNs and their complex regulation in these organisms. We review here this “alternative” fish IFN system, which evolved in parallel to the one known in tetrapods.

## 2. Fish Type I Interferons: An Astonishing Diversity

### 2.1. Structural Diversity of IFN Sequences

The first fish type I IFN sequences were identified in 2003 from genomic data by B. Robertsen and his collaborators in Atlantic salmon (*Salmo salar*), by the group of C. Kim in zebrafish (*Danio rerio*), and by G. Lutfalla in pufferfish (*Tetraodon nigroviridis*) [9,10,11]. These genes showed relatively weak sequence similarity with mammalian type I IFNs, which explained the failure of homology cloning trials, and were made of five exons and four introns, while mammalian IFNα and IFNβ are intronless genes. IFNs later found in cartilaginous fish [12] and frogs [13] share the same structure as those of teleosts, with introns, which suggests that the ancestor of type I IFN genes contained introns, that were lost (likely by retroposition) during the evolution of Amniotes.

Transfection with fish IFN genes or treatment with the recombinant proteins showed a strong antiviral activity in cell culture, assayed by inhibition of virus plaques and/or induction of *MX* gene expression [9,10,11].

**Figure 1:**
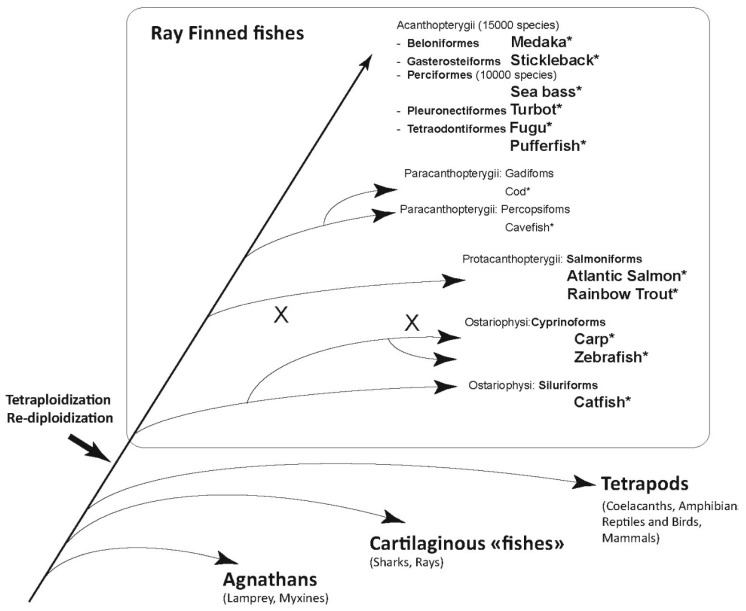
Schematic phylogenetic tree of fishes. Taxonomy from [20]. The thick black arrow indicates a whole genome duplication (WGD) during the early evolution of ray-finned fishes. X indicate examples of posterior WGD in specific branches. Genome sequence is available for all species indicated with *. Species in bold letters are mentioned in Table 1.

Variable numbers of genes were discovered in different fish species during the following years (reviewed in [14], see Figure 1 and Table 1), which were classified into two main subgroups based on the number of cysteine bridges they contain [15]. Group I comprises IFN sequences with two conserved cysteines (C1 and C3) forming a disulfide (S-S) bridge, while group II sequences have four conserved cysteines and two conserved S-S bridges (C1/C3, and C2/C4), as confirmed in [16]. Four cysteine (4C) containing sequences are found across all vertebrates from sharks to mammals, while two cysteine (2C) containing sequences have also been found in teleost genes (in which C1 and C3 would make a S–S bond); a few mammalian sequences (like IFNβ and -ε) also have only two conserved cysteines, corresponding to the C2/C4 S-S bond [15]. Additional Cs are observed at non conserved position in IFNs of some species like Turbot, which likely are not involved in S–S bridges [17].

The variation of IFN gene numbers between fish species is impressive (Table 1). The most complex system is probably the one described in salmonids. With about forty open reading frames (ORFs) identified via bacterial artificial chromosome (BAC) clone analysis, the type I IFN repertoire of rainbow trout (*Oncorhynchus mykiss*) outnumbers ours by a factor of at least two. These genes fall into three 2C subgroups (a, d, and e), and three 4C subgroups (b, c, and f) [18]. In Atlantic salmon, 11 IFN genes have been identified but there are certainly more [19].

The diversity found in other teleosts appears to be smaller, according to the current sequence data; only the subgroups a, c and d seem to be present.

Cyprinids possess the three subgroups (a, c and d). In zebrafish (*Danio rerio*), for which a high quality genome is available, four type I IFNs were identified, falling into group I/2C (IFNφ1, subgroup a, and IFNφ4, subgroup d) and group II/4C (IFNφ2 and φ3, subgroup c) [15,21]. The repertoire of type I IFNs found in common carp is more complex, with approximately twice as many genes as in zebrafish. In channel catfish (*Ictalurus punctatus*), which also belong to Ostariophysi, only group I (2C) IFNs have been described so far [10,22].

For a long time, it also seemed that only group I IFNs were present in the most diverse group of teleost fish, the Acanthomorphs. Medaka (*Oryzas latipes*) has one IFNa and one IFNd, and stickleback (*Gasterosteus aculeatus*) has one IFNa and three IFNd. Acanthopterygians (perch, flatfish, pufferfish, etc.) often have only the d subgroup (possibly with multiple gene copies) [10,22]. However, the large branch of Percomorphs does not entirely lack group II IFNs, since turbot has one IFNc (named IFN1) and one IFNa (named IFN2) [17,23]. In fact, significant sequence information is available only in a few fish species, and the great diversity of this group may hold surprises in store.

### 2.2. Nomenclature Issues

Several research groups working on different fish families have established different nomenclatures (Table 1, reviewed in [14]). With new groups of type I IFNs recently added to the list [18], the classification of fish type I IFNs has become more complex and rather confusing. Thus, group I (with 2C) comprises subgroups a, d, and e, while group II (with 4C) comprises subgroups b, c and f, which is certainly not easy to remember. Things get worse when confronted to the mammalian nomenclature: salmonid fish IFNa, IFNb or IFNe are wrongly reminiscent of mammalian IFNα, β and ε. To avoid this problem, the cyprinid/zebrafish nomenclature introduced by Stein et al. [25] includes a φ (for “fish”) but has other inconsistencies with IFNφ1 and φ4 in group I, and IFNφ2 and φ3 in group II, with no obvious correspondence with the subgroups defined in salmonids. In addition, IFN1 and 2 from other species may fall in any group, with both IFN1 and IFN2 from catfish falling in group I (2C), while in Turbot, IFN1 is in group II (4C) and IFN2 in group I.

The perfect nomenclature should be practical, simple, self-explanatory, consistent with available and future data, and associated to a clear definition of each category. Ideally, it should also clarify that the subgroups found in fish are not orthologous to those known in mammals. The nomenclature in mammals was also confusing for a while; in 1980, an article providing recommendations for IFN nomenclature compared the “new” nomenclature (IFNα, -β, and -γ) with the “old” nomenclature in which IFNα was called leukocyte (le), fast (F) or C and IFNβ fibroblast (F), slow (S) or A/B [26]. It was recommended for other species to make preliminary classifications based on antigenic homology using antisera against human or mouse IFNα and IFNβ. Whether Greek letters or other symbols should be used remains open, but it is time for fish IFN immunologists to create a unified and easier nomenclature that would avoid confusion with mammalian denomination.

### 2.3. Fish Type I Receptors: Several CRFB Complexes

**Figure 2:**
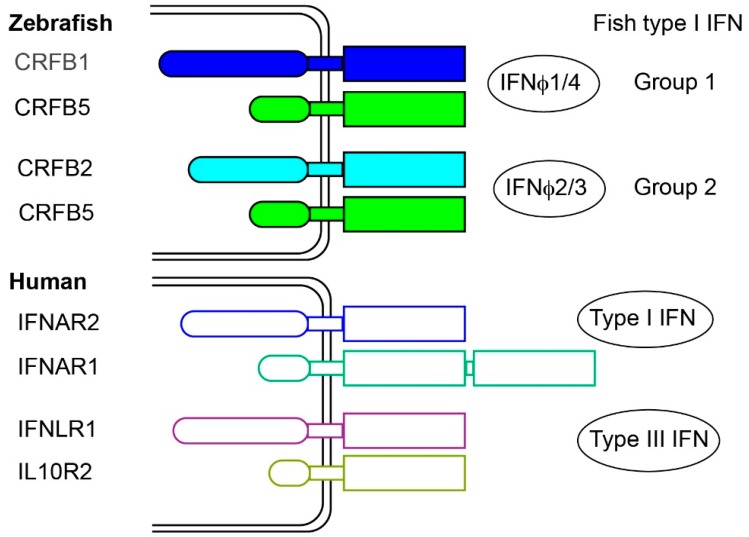
Receptors of virus induced IFN in zebrafish and human. Adapted from [21]. Intracellular regions are represented by round boxes, and D200 domains by rectangular boxes. IFN binding to each receptor are represented on the right.

Type I IFN receptors are typically heterodimers of members of the class II receptor (CRFB) family. Identifying CRFB family genes from fish sequence data was straightforward, based on the presence of an extracellular D200 domain, but identifying the IFN receptors among them was not [10]. Using the zebrafish model, *crfb* candidate genes were screened by in vivo experiments. Zebrafish embryos were injected with a plasmid encoding IFNφ1 (group I, 2C), and with morpholinos knocking down each specific *crfb*. Recombinant IFNs induced a robust expression of the ISG viperin, which was strongly down-regulated in the absence of the IFN receptor. Using this system, and reciprocal in vivo gain-of-function assays, *crfb1* and *crfb5* were identified as the genes encoding the two chains of the IFNφ1 receptor [27]. The same approach with IFNφ2 or IFNφ3 (group II, 4C) identified *crfb2* and *crfb5*, while the weak response induced by IFNφ4 (group I, 2C) relied on *crfb1* and *crfb5*, indicating that fish IFNs from group I and II use different receptors [21]. This was in accordance with competition experiments performed in rainbow trout using recombinant IFN, indicating that group I and II IFN did not share the same receptor complex [15]. CRFB1 and CRFB2 have one D200 ligand binding domain, and a long intracellular tail, as mammalian IFNAR2; the other chain, CRFB5, likely playing the role of IFNAR1, has also a short intracytoplamic region but a single D200 domain instead of two as in IFNAR1. Thus, the structure of the fish IFN receptor appears more similar to the mammalian IFNλ receptor, made of two chains with a single D200, than to the typical IFNAR complex. Thus, both the five-exon gene organization and the receptor structure of fish IFNs differ from that of mammalian type I IFNs but resemble that of mammalian IFNλs, which led to the proposition that fish IFNs (and the ancestral vertebrate IFNs) were type III IFNs [27] (Figure 2). However, this hypothesis was definitively ruled out by the crystallography of zebrafish IFNφ1 and IFNφ2, which demonstrated their typical type I IFN nature [16]. In fact, type III IFNs appear to be absent in teleost fish.

A large repertoire of CRFB has been described in the Atlantic salmon, with two clusters located on different chromosomes; these genes might be involved in an additional diversity of receptors matching the complexity of IFN molecules in salmonids [28].

### 2.4. Multiple Transcripts and Intracellular IFNs

**Figure 3:**
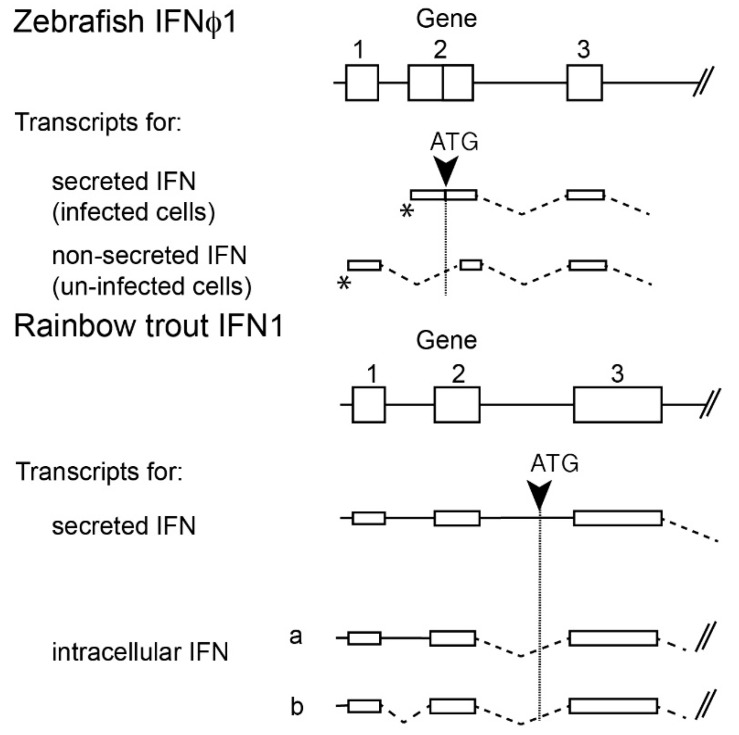
Different mechanisms lead to transcripts encoding secreted and non secreted IFN (group I). Exons (numbered 1–3) are represented as boxes. In transcripts, spliced introns are represented by dotted lines, while plain lines correspond to un-spliced introns. ATG corresponding to the secreted IFN open reading frame (ORF) are indicated by a black arrow. * denote alternative transcription start points in zebrafish. Note that mechanisms leading to production of mRNA encoding secreted IFN are different in zebrafish and rainbow trout (alternative transcription start versus alternative splicing). Adapted from [27,30,31].

Mammalian type I IFNs are typically secreted after induction by viral infection, and act in autocrine or paracrine manner via IFN receptor located at the cell external membrane. Fish IFNs of the IFNa/IFNφ1 subgroup have a more complex regulation. Their promoter is typically made of two regions, and an alternative use of initiation of transcription leads to different transcripts, as discovered in Atlantic salmon [29]. In zebrafish, the long transcript of IFNφ1 is constitutively expressed at a fair level, and poorly inducible; in contrast, viral infection induces the use of the alternative promoter, leading to a shorter, highly inducible transcript; importantly, the “long” transcript does not contain a signal peptide and therefore is apparently not secreted, while the “short” inducible transcript is efficiently secreted [27]. Similar mechanisms have also been observed in rainbow trout [30] (Figure 3). While the function of the long transcripts leading to non-secreted IFN was initially elusive, it was later observed that their overexpression leads to induction of *Mx* and to a protection against viral infection [31]. Remarkably, CRFB genes encoding the receptor also have alternative transcript variants lacking signal peptide; hence, these receptors are probably absent from the cell membrane, but seem to be mainly located in the perinuclear region, where they could co-localize with the intracellular IFN molecules. As overexpression of both intracellular IFNs and receptors leads to STAT signalling and induction of resistance to viral infection, it is proposed that they act as a partly constitutive cellular system of antiviral defence [31]. Intracellular IFNs may also act as alarmins, being released upon cell lysis and activating neighbouring cells after binding their surface receptors, although experimental evidence for such a pathway is still lacking.

### 2.5. Evolutionary Pathway of Type I IFN

**Figure 4:**
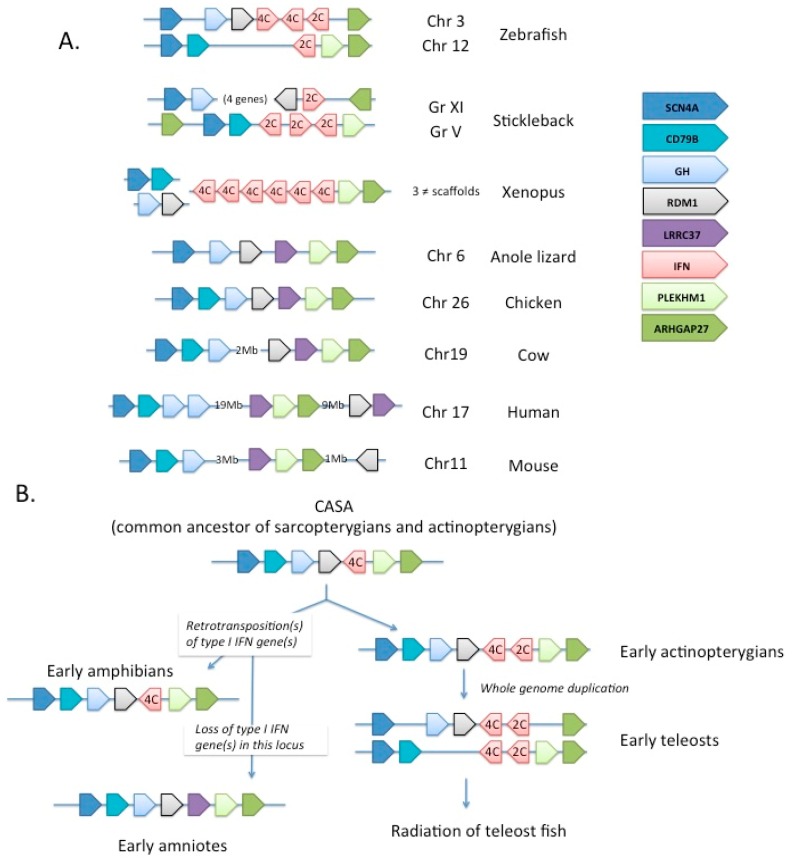
Evolution of the type I IFN locus. (**A**) Current structure of *scn4a/ahrgap27* loci in various vertebrate genomes, illustrating the loss of type I IFN gene in this region likely from the common ancestor of amniotes. The color code for the different markers of this synteny group is represented on the right of the panel. IFNs of the four- and two-cysteine types are noted as 4C and 2C; (**B**) Tentative reconstitution of the evolution of this region in Vertebrates, illustrating the presence of type I IFN genes in the *scn4a/ahrgap27* locus from the common ancestors of fish and tetrapods, and their loss during the evolution of Amniotes.

Phylogenetic analyses and studies on the intron/exon structure of type I IFN genes indicate that the ancestor of type I IFN in early vertebrates was likely a class II helical cytokine gene with introns and four cysteines; the variable features observed between groups of vertebrates are likely derived [13,14,27]. Thus, the intronless IFN genes found in amphibians, reptiles (including birds) and in mammals would have originated in retroposition event(s), which likely occurred during the early evolution of tetrapods, and the different patterns of C and S–S bonds have evolved in parallel in fish and in tetrapods. Interestingly, the large diversity of type I IFN genes observed in several groups like salmonids, some cyprinids, and mammals, has been produced independently and seems to be the result of a convergent evolution. A diverse array of type I IFN genes should constitute a beneficial trait, likely through functional specialization of the multiple copies or ability to escape virus inhibition (see below, Part 2). This view is also supported by the recent discovery of expanded intronless type I (and type III) IFN in frogs, besides type I and type III IFN genes with introns as found in fish [13,32]. Thus far, no intronless type I IFN genes have been found in fish, and all type I IFN genes identified in the coelacanth genome have introns [33]. Overall, these observations suggest that individualization of type I and Type III IFN, as well as intron loss by retro-transposition happened relatively early in the tetrapod evolution, predating the last common ancestor of frogs and mammals. In fact, retro-transposition events and independent expansions might have occurred several times (Figure 4).

Other clues of evolution of type I IFNs can also be deduced from synteny analysis (Figure 4B). In zebrafish, the *ifnphi1*, *-2* and *-3* genes are found in tandem on chromosome 3, while *ifnphi4* is on chromosome 12 (www.ensembl.org). Clearly, the two loci arose from the teleost-specific whole genome duplication (WGD) event, as they are flanked by paralogous genes with a single homologue in tetrapods (*SCN4A* on one side, *ARHGAP27* on the other). Interestingly, no IFN gene is found linked to these genes in the genomes of anole lizard or chicken (where the two genes are also closely linked), or in mammals (where they are distant), but type I IFN genes (with introns) are found next to *ARHGAP27* in Xenopus, implying that the *SCN4A*/type I IFN (with five exons and 4C)/*ARHGAP27* configuration was already present in the last common ancestor of teleosts and tetrapods. The loss of type I IFN at this position in amniotes fits perfectly with the hypothesis of a decay of the original five-exon IFN genes, while intronless genes produced by retro-transposition expanded and replaced them completely. Interestingly, an unrelated *LRRC37* gene is now found in this position in amniotes, which is probably just a coincidence.

The two loci in zebrafish contain (2C 4C 4C) and (2C) type IFNs, respectively. Since IFN genes with 4C appear ancestral, the most parsimonious hypothesis would be that the original 4C gene had a tandem duplication, giving rise to a 2C neighbour, prior to the teleost WGD, endowing early teleosts with (2C 4C) and (2C 4C) loci, which would then have further evolved in the diversity of type I IFN repertoires in the different fishes. The diversification would have occurred via multiple events of local duplications and WGD, depending of fish group. How rapidly these genes evolve and sub-functionalize after WGD will be very interesting to study when more data about the role of each gene will be available.

## 3. Differential Expression and Functional Properties of Fish Type I IFNs

Why do fish, like humans, have so many type I IFNs? Duplication and divergence of such important immune genes is probably fuelled by the need to escape inhibition by some viruses. It also allows for more functional diversification, either in expression (in location and/or timing) or in downstream effects. Our knowledge is still very limited, but variation on both expression and function is seen among fish IFNs, some of which even appear to predate the teleost/tetrapod split despite the independent diversification of the genes in the two lineages.

### 3.1. Expression

#### 3.1.1. Cyprinids

In zebrafish, IFNφ1 (secreted form) is expressed at very low levels in the uninfected larva, but at a significant level in the adult spleen. It is well induced by viral infection in both cases. IFNφ2 is not expressed (and not inducible) in larvae, while the expression in adults matches that of IFNφ1. IFNφ3 is expressed at relatively high constitutive levels in both larvae and adults, but is still inducible by viral infection. IFNφ4 is expressed at low level both in larvae and adult spleen, and is only weakly inducible [21]. A transgenic reporter zebrafish identified hepatocytes and neutrophils as the two main cell types expressing IFNφ1 (group I) in larvae upon alphavirus infection [34]; a more recent IFNφ3 (group II) reporter line suggests a completely non-overlapping pattern of expression, including fibroblasts (Briolat, Lutfalla, and Levraud, unpublished result). The contributions of IRF1/3/7 to the induction of IFNφ1 and IFNφ3 have been studied in vitro, providing first insights for anatomical complementarity of different IFN [35], as detailed below. In grass carp (*Ctenopharyngodon idella*), four type I IFN were also identified, CiIFN1 and four with two cysteines (group I) and CiIFN2 and three with four cysteines (group II); group II IFNs were transiently induced in gills and spleen by GCRV infection in adult fish, while CiIFN1 was constitutively expressed in gills, spleen, liver and gut, and CiIFN4 was never detectable in any condition tested [36].

#### 3.1.2. Salmonids

In rainbow trout, a first analysis of tissue specific expression profiles revealed clear differences between IFN1, IFN2 and IFN3 genes (group II) [15]: IFN2 (also known as IFNa2, group I) was detected in all analysed tissues, namely brain, gill, gut, kidney, liver, muscle, skin, spleen, ovaries, and testes; in contrast, IFN1 (also known as IFNa1, group I) was never expressed in the studied tissues, while IFN3 (also known as IFNb1, group II) was expressed in ovaries and testes, albeit at a lower level than IFN2, and was barely detectable in brain, gut, muscle, and skin. All these genes were inducible by polyI:C or viral infection [15]. Later studies with members of all subtypes [18] showed that group I IFNs were expressed in untreated RTG2 (fibroblastic) and RTS11 (macrophage-like) cell lines, and in head kidney leukocytes ex vivo. Stimulation of RTG2 and RTS11 cell lines by polyI:C revealed again disparities even within groups: group I IFN often showed strong and fast induction, with IFNd group I showing the fastest induction; in contrast, IFNb and c (group II) were still not induced 2 h post stimulation. Interestingly, IFNf (group II) was induced fast and well by poly I:C, as IFNa and d. In head kidney leukocytes, ex vivo polyI:C treatment induced all subtypes, with IFNa, b and c showing the strongest up-regulation. Interestingly, treatment with recombinant IFNa2 (also known as IFN2, group I) induced IFNa and IFNd in RTS11 cells, showing that auto-amplification of type I IFN response is possible in some conditions, while it is not a typical feature of bird or mammal type I IFN.

The induction patterns of all subtypes were also studied in brown trout (*Salmo trutta*) during viral hemorrhagic septicemia virus (VHSV) infection in vivo, in spleen and head kidney [18]. The most strongly induced IFN genes were IFNa (group I) and IFNb and c (group II), as soon as three days post infection and remaining expressed for more than 10 days. IFNd and -e (group I) were moderately upregulated, only in the early stages of the disease. IFNf (group II) was induced very early and its expression increased until day 7. Taken together, these data were in contrast with the (more) simple situation reported in zebrafish, in which group II IFN were induced early and transiently, in contrast to group I IFNs [37].

In Atlantic salmon, IFN subtypes a, b and c were found to be constitutively expressed in head kidney, but at very different levels [19]: for example, IFNa1 and IFNc transcripts were expressed more than 50 times greater than IFNa3 and IFNb. Upon in vivo treatment by polyI:C, IFNa was induced during the first 24 h then declined, while IFNb was not induced and IFNc levels increased until 96 h post stimulation; in contrast, treatment with an imidazoquinoline derivative (a TLR7 agonist) led to transient induction of IFNc, sustained induction of IFNa and very strong although short upregulation of IFNb.

These multiple studies revealed a considerable diversity of expression and induction patterns of salmonid IFN genes, but consistently underlined the importance of IFNa and IFNc responses, likely in a cell type specific manner. This pattern was confirmed in another study comparing IFNa, b, c and d in Atlantic salmon [38]. The antiviral activity of recombinant proteins against infectious pancreatic necrosis virus (IPNV) in the macrophage like cell lines TO was contrasted: IFNa and c had a strong activity, IFNb a significant but much lower activity (>500 times), and IFNd showed no antiviral activity at all against IPNV. This profile was consistent with the rainbow trout data regarding IFN1, IFN2 versus IFN3 from [15] (see above). Induction experiments confirmed that IFNa on the one hand and IFNb and -c on the other hand use different main induction pathways, while IFNd was apparently not induced by poly I:C or imidazoquinoline. Importantly, FISH) experiments revealed that poly(I:C) induced IFNa and IFNc in various cell types in lymphoid and non lymphoid tissues, while the imidazoquinoline induced both IFNb and IFNc in particular cells in spleen and head kidney. Such cells are likely very high IFN producers: indeed, while a small number is apparently present, it should account for the whole IFNb and -c synthesis. These data provided the first evidence for specialized IFN producing leukocytes in fish (however, there is, as yet, no evidence for or against the presence of plasmacytoid dendritic cells in fish); interestingly, such cells do not secrete all type I IFN subtypes, which is reminiscent of the IFNα versus IFNβ production pathways in mammals. The cell specific expression of the numerous salmonid IFN subtypes remains to be determined, as well as the different patterns of expression found across teleosts.

If one compares expression patterns of IFNs in cyprinids and salmonids, significant differences (beyond the number of genes) are noted; for example, the IFNs which seem to be specifically expressed by leukocytes belong to group I IFNs in zebrafish and group II in salmon. However, differences in life stages analysed and the kinetics of induction have to be kept in mind, so it is too early to draw conclusions on this point.

### 3.2. Control by IRFs

IRFs, especially IRF3 and IRF7, are the main transcription factors controlling IFN expression [39]. In mammals, IFNβ transcription is triggered by binding of NF-κB and by IRF3/IRF7 heterodimers to its promoter, but also by IRF3 only, which plays a key role in early responses, since IRF3 is expressed ubiquitously and not increased by IFN signalling, while IRF7 is IFN-induced; by contrast, IFNα expression requires IRF7 homodimers and is largely IRF3-independent. This simplified framework omits the role of many other transcription factors (TFs) (such as IRF1) that also contribute to IFN expression, but has general explanatory power; for instance, the constitutive expression of IRF7 by plasmacytoid dendritic cells (pDCs) explains why these cells secrete 1000-fold more IFNα than other cells, on a per-cell basis, during the early phases of viral infections.

Both IRF3 and IRF7 have a unique orthologue in all fish genomes fully sequenced. There is also a single IRF1 orthologue, although the early nomenclature has been confusing (the proper zebrafish gene has been named first *irf11* then *irf1b*; [40]). A significant divergence between fish and mammals is that all three genes are IFN-induced in fish, while this is only the case for IRF1 and IRF7 in mammals [41]. The requirements for IRFs in fish IFN expression has been investigated by the group of Jian-Fang Gui using luciferase reporter assays with fragments of zebrafish IFN promoter sequences. By overexpressing wild-type or dominant-negative (DN) versions of fish IRFs (from crucian carp, or later from zebrafish), it was found that IRF3 alone was sufficient to induce IFNφ1 but not IFNφ3 reporter constructs, while IRF7 alone induced the IFNφ3 promoter construct, but more weakly an IFNφ1 construct. For both promoters, higher expression was achieved by co-expression of both IRFs. IRF1 could also activate both reporters (IFNφ3 better than IFNφ1) and synergized with either IRF3 or IRF7. When the reporters were activated by an “upstream” trigger (such as overexpression of TBK-1), expression of a dominant negative version of either IRF3 or IRF7 (but not IRF1) inhibited luciferase production [40,42]. Further biochemical studies indicated that these three IRFs can either homo- or hetero-dimerize and suggested that IRF3 facilitates the binding of IRF1 or IRF7 to the promoters [35]. In summary, the zebrafish IFNφ1 promoter is predominantly controlled by IRF3, and IFNφ3 by IRF7, like IFNβ and IFNα, respectively. Interestingly, this does not fit easily with the cell-specific expression profiles of these genes in vivo as revealed by reporter transgenes (see above), with IFNφ1 being expressed by leukocytes (reminiscent of IFNα) and IFNφ3 by stromal cells (more like IFNβ). Further study is needed, but clearly one should be very cautious, as the subgroups of type I IFNs of fish and mammals do not seem to be functionally matched in a simple way.

### 3.3. Functions

#### 3.3.1. Cyprinids

In zebrafish, overexpressed or recombinant IFNφ1 could protect efficiently larvae or adults against different viruses including snakehead rhabdovirus, infectious hematopoietic necrosis virus (IHNV) and spring viraemia of carp rhabdovirus (SVCV) [11,21,27,37,43]. IFNφ2 or IFNφ3 also protected against SVCV or IHNV (22, 31). IFNφ4, however, did not protect embryos against IHNV infection, which was correlated with a much weaker ISG upregulation (22). The antiviral activity was well correlated to the capacity of ISG upregulation, IFNφ1 and -2 being potent inducers of viperin while IFNφ4 being a weak one. IFN genes also have contrasted expression and induction patterns that differ between larvae and adults. In the adult, IFNφ2 and IFNφ3 induced a rapid and transient expression of ISG (measured in the head of the fish), while IFNφ1 induced both ISG and inflammatory genes, more slowly but at higher levels. These observations suggest complementary functions of different IFN, which also possess distinct activities against viral and bacterial pathogens [37].

Type I IFNs are well known for their antiviral activities, but may also impact bacterial infections, in either a positive or a negative way [44]. So far, this activity has not been extensively characterized in fish, but interestingly, it has been found that in zebrafish, the group I IFN IFNφ1, but not the group II IFNs IFNφ2 or IFNφ3, could confer protection against a challenge with *Streptococcus Iniae* [37]—although this bacterium did not induce by itself a measurable IFN response. By contrast, *Salmonella typhimurium* of *Mycobacterium marinum* have been shown to (modestly) induce IFNφ1 expression in zebrafish larvae, by different pathways [45] although it is unclear if this impacts the infection course.

#### 3.3.2. Salmonids

Differences in efficiency have been found among salmonid IFNs. For example, recombinant IFNa1 and IFNa2 induced *Mx* and protected RTG2 cells against VHSV, while IFNb1 did not [15]. One of the most intriguing observations about fish type I IFNs bioactivity relates to the effect of injection of plasmids encoding type I IFNs. Plasmids expressing salmon IFNa1, IFNb or IFNc were co-injected with plasmid encoding the hemagglutinin-esterase (HE) gene of infectious salmon anaemia virus (ISAV) to presmolts [46]. While the vaccine plasmid alone induced low antibody titres and led to a weak protection against ISAV infection, all IFN tested provided a strong adjuvant effect, affording good protection with higher virus specific IgM titres [46]. The mechanisms of this adjuvant effects remain elusive, although attraction of lymphocytes to the sites of injection was reported [46]. Additionally, remarkable differences have been observed when testing the in vivo antiviral activity of intramuscular (i.m.) injection of plasmids encoding IFNa1, IFNb or IFNc not associated with HE plasmid [47]. All three IFN induced typical ISG locally, close to injection site. However, only IFNb and IFNc induced ISG expression in head kidney, liver and heart of injected fish, suggesting that IFNb and IFNc can be active systemically, while the IFNa1 effect is restricted to the injection site. Additionally, IFNc plasmid induced a long lasting expression of RIG-I, TLR3 and TLR7 in head kidney, over several weeks. When infected by ISAV eight weeks after injection of IFN plasmids, fish were strongly protected by IFNc, weakly protected by IFNb, and not at all by IFNa1. Interestingly, the expression of the ISG *Mx* was assessed on sections of heart and liver at this time point, and was higher in fish injected with IFNc plasmid, compared to IFNb.

Overall, the type I IFN system of salmonids is a remarkable model to study subfunctionalization of a cytokine group, but more viral models and more complete characterization of ISG induction profiles will be necessary to get a global understanding of the complementarity between subgroups and their members.

#### 3.3.3. Other Fish Groups

In medaka, the respective bioactivities of IFNa and IFNd were assessed after transfection in the medaka hepatoma cell line DIT [23]. IFNa upregulated IFNd expression, and vice versa; both IFN induce typical ISG and could protect transfected cells against nervous necrosis virus (NNV), apparently with a similar efficiency. In turbot, injection of a plasmid expressing IFN1 (group II), but not of a plasmid expressing IFN2 (group I), led to induction of typical ISG and protection against VHSV infection [17]. Overall, the large diversity of perciforms has not been well explored yet; interestingly, a recent report in the yellow croaker confirmed that the distinction between group I and group II IFN also hold for this vast group [48], although genome contraction as in tetraodontiforms, or additional duplications, certainly led to large variations of the repertoire.

## 4. The Effectors of Fish Type I IFN: Parallel Evolution of Fish and Mammal ISG Genes and Families, Regional Expression and Regulation in Ontogeny

Vertebrates share a common, generic set of immune genes. However, since a whole genome duplication occurred during the early fish evolution [49,50], the repertoire of fish immune genes is potentially more diverse, compared to the one of mammals. Indeed, pairs of duplicated genes provided opportunities for sub-functionalization and specialization. Additionally, many lineage specific duplication events, for example in salmonids and cyprinids, further increased this genomic diversity [51]. ISG are no exceptions.

Early studies of fish ISG identified conserved fish genes induced by virus infection, like *Mx* [52], and confirmed that they were upregulated by type I IFN in fish as well as in mammals. Differential approaches such as differential display or subtraction-suppression-hybridization between transcriptomes from control and virus-infected tissues identified the most expressed ISGs [53,54,55,56], comprising a few fish specific genes but a large majority of ISG conserved across vertebrates. These studies showed that in fish, as in mammals, the main transcriptional response induced by viral infection is based on (conserved) ISGs [55].

With the development of micro arrays and high-throughput sequencing, the description of fish ISG became more comprehensive. For example, the transcriptional response to the alphavirus chikungunya, which is mainly based on ISG upregulation, was recently characterized in the zebrafish embryo [57]. A core set of ISG conserved between zebrafish and human could be defined, in contrast to multigenic families diversified independently in fish and mammals. Thus, zebrafish orthologs of the exhaustive ISG list compiled from human studies by Schoggins et al. [7] were strongly enriched in genes modulated during IFN response, and it was estimated that zebrafish possesses about 100 IFN induced genes among the orthologs of human ISGs [57]. The case of multigenic families is particularly interesting, with several evolutionary patterns. Some “old” families have already diversified in the common ancestor of fish and mammals and did not diversify in parallel in each lineage; in this case, each human ISG had a single orthologue (or a pair of orthologs due to the whole genome duplication) in zebrafish, which is also IFN inducible. This is typical of regulatory components like transcription factors or proteins involved in signal transduction. More “recent” families have evolved and diversified in parallel in fish and tetrapods, likely under strong diversifying pressures. These families, including for example *Mx* and *IFIT*, often comprise both ISGs and non ISGs. The tripartite motif protein family (TRIM), of which many members play key roles in antiviral immunity in fish and mammals [58], is a good example of this evolutionary pattern. Most TRIM with a key role in mammalian antiviral defense, such as *TRIM5* [59], *TRIM22* [60], *TRIM21* [61], *TRIM19* [62], etc. do not have a direct orthologue in fish [63]. In contrast, a large TRIM subset of more than 80 members is found in the zebrafish genome, with all the hallmarks of an implication in immunity such as evolution under positive selection, but without equivalents in mammals [64,65]. Interestingly, this fish specific subset is absent from the coelacanth, which is a representative of an early branch of tetrapods; this species possesses its own set of diversified TRIM genes [33]. There are, however, exceptions such as TRIM25 that is conserved across vertebrates from fish to humans, and probably has had a primordial role in type I IFN induction pathways [63,65].

The zebrafish model also revealed that ISGs were expressed with a tissue restricted pattern, suggesting that all cell types may not equally express type I IFN receptors. Most ISGs studied showed a strong induced expression in the liver, gut and blood vessels [57]. While there was some overlap with the expression pattern of IFNφ1 [34], with a strong level in the liver, the respective contribution of the different type I to the modulation of the ISG repertoire remains unknown. The combination of IFN genes, with their own expression patterns and receptor pathways, should determine the effector response, and likely depends on viruses or stimulation contexts.

As in humans, the maturation of the type I IFN response determines largely the sensitivity to virus infections during the early life stages. In humans, four main phases of development of the immune system have been identified [66]: before birth, a phase with low immunity dominated by anti-inflammatory cytokines such as interleukin (IL)-10, around birth, a phase in which leukocytes produce large amounts of IL-6 and IL-23 upon toll-like receptor (TLR) stimulation, but almost no type I IFN; type I IFN responsiveness matures a few weeks after birth, while pro-inflammatory and Th1 responses develop progressively over the first years of life. Distinct phases were also observed when the transcriptional response to VHSV infection was analysed during the critical transition of rainbow trout embryos to fry. The type I IFN response was very low, with a limited number of responsive genes, at eyed eggs and hatching stages. A clear IFN response was detected only after first feeding and matured over the following weeks [67]. While hatching is an important change for the fry, bringing closer contact with the environment, the opening of the mouth and first feeding represents a critical transition for the fish immune system: the gut microbiota has to get established, and the interactions with the environment especially the pathogens, intensify. It will be interesting to dissect the respective kinetics of inducibility for different IFN genes, and the impact on the upregulation of distinct associations of ISGs by various viruses. Such data will provide new insights into the maturation of higher resistance to viral infections.

## 5. Conclusions and Perspectives

Fish type I IFN display an extraordinary diversity, which likely represents an adaptation to multiple viral strategies to evade the innate immunity of the host. Only a few families have been investigated in detail, either because they are important in aquaculture or because they constitute popular models, such as zebrafish; new features will likely be revealed by future studies of genomes from the large group of Percomorphs, and also from old branches of fish. The functional specificity of individual IFN genes (expression pattern, repertoire of ISG, as well as antiviral, adjuvant and immuno-regulatory activities) remains poorly known, but deserves further investigation.

## Figures and Tables

**Table 1:** Phylogenetic relationships between fish type I interferons (IFNs); from [17,18,23,24].

	Rainbow Trout *Oncorhynchus mykiss* (Salmonid)	Atlantic Salmon *Salmo salar* (Salmonid)	Zebrafish *Danio rerio* (Cyprinid)	Channel Catfish *Ictalurus punctatus* (Ictalurid)	Medaka *Oryzias latipes* (Percomorph)	Turbot *Scophthalmus maximus* (Percomorph)
**Group I (2C)**						
Sub Group a						
	IFNa1 (=IFN1) (NM 001124531) IFNa2 (=IFN2)( NM 001160505) IFNa3 (HF931021) IFNa4 (HF931022)	IFNa1 (NM 001123710) IFNa2 (NM 001123570) IFNa3 (EU768890)	IFNφ1 (=IFN, IFN1) (NM_207640.1)	IFN1 (AY267538) IFN2 (AY847295) IFN4 (AY847296)	IFNa (LC066594)	IFN2 (KJ150678)
Sub Group d						
	IFNd1 (=IFN5) (NP_001152811)	IFNd1 (NM_001279092.1)	IFNφ4 (?) (NM_001161740.1)		IFNd (LC066595)	
Sub Group e						
	IFNe1-7 (HF931030-6)					
**Group II (4C)**						
Sub Group b						
	IFNb1 (=IFN3) (NP_001153974) IFNb2 (=IFN4) (NP_001158515) IFNb3 (HF931023) IFNb4 (HF931024) IFNb5 (HF931025)	IFNb1 (EU735552) IFNb2 (EU768890) IFNb3 (EU768890) IFNb4 (EU768890)				
Sub Group c						
	IFNc1-4 (HF931026-9)	IFNc1-3	IFNφ2 (NM_001111082.1) IFNφ3 (NM_001111083.1)			IFN1 (KJ150677)
Sub Group f						
	IFNf1-2 (HF931037-8)					

Note that within a Subgroup, genes mentioned for a given species are (as a group) co-orthologs of the gene/gene sets indicated for the other species. For a complementary list of ID, see [14]. Regarding interferon φ4 (IFNφ4), the “?” reminds that bootstrap values in phylogenetic analyses do not fully demonstrate that IFNφ4 is an orthologue of IFNd from other species.

## References

[1] Zou J., Secombes C.J. (2016). The Function of Fish Cytokines. Biology (Basel).

[2] Isaacs A., Lindenmann J. (1957). Virus interference. I. The interferon. Proceedings of the Royal Society of London.

[3] Taniguchi T., Mantei N., Schwarzstein M., Nagata S., Muramatsu M., Weissmann C. (1980). Human leukocyte and fibroblast interferons are structurally related. Nature.

[4] Taniguchi T., Fujii-Kuriyama Y., Muramatsu M. (1980). Molecular cloning of human interferon cDNA. Proc. Natl. Acad. Sci. USA.

[5] Antonelli G., Scagnolari C., Moschella F., Proietti E. (2015). Twenty-five years of type I interferon-based treatment: A critical analysis of its therapeutic use. Cytokine Growth Factor Rev..

[6] Schoggins J.W., MacDuff D.A., Imanaka N., Gainey M.D., Shrestha B., Eitson J.L., Mar K.B., Richardson R.B., Ratushny A.V., Litvak V. (2014). Pan-viral specificity of IFN-induced genes reveals new roles for cGAS in innate immunity. Nature.

[7] Schoggins J.W., Wilson S.J., Panis M., Murphy M.Y., Jones C.T., Bieniasz P., Rice C.M. (2011). A diverse range of gene products are effectors of the type I interferon antiviral response. Nature.

[8] De Kinkelin P., Dorson M. (1973). Interferon production in rainbow trout (*Salmo gairdneri*) experimentally infected with Egtved virus. J. Gen. Virol..

[9] Robertsen B., Bergan V., Røkenes T., Larsen R., Albuquerque A. (2003). Atlantic salmon interferon genes: Cloning, sequence analysis, expression, and biological activity. J. Interferon Cytokine Res..

[10] Lutfalla G., Crollius H.R., Stange-Thomann N., Jaillon O., Mogensen K., Monneron D. (2003). Comparative genomic analysis reveals independent expansion of a lineage-specific gene family in vertebrates: The class II cytokine receptors and their ligands in mammals and fish. BMC Genom..

[11] Altmann S.M., Mellon M.T., Distel D.L., Kim C.H. (2003). Molecular and functional analysis of an interferon gene from the zebrafish, Danio rerio. J. Virol..

[12] Chang M., Nie P., Collet B., Secombes C.J., Zou J. (2009). Identification of an additional two-cysteine containing type I interferon in rainbow trout Oncorhynchus mykiss provides evidence of a major gene duplication event within this gene family in teleosts. Immunogenetics.

[13] Qi Z., Nie P., Secombes C.J., Zou J. (2010). Intron-containing type I and type III IFN coexist in amphibians: Refuting the concept that a retroposition event gave rise to type I IFNs. J. Immunol..

[14] Zou J., Secombes C.J. (2011). Teleost fish interferons and their role in immunity. Dev. Comp. Immunol..

[15] Zou J., Tafalla C., Truckle J., Secombes C.J. (2007). Identification of a second group of type I IFNs in fish sheds light on IFN evolution in vertebrates. J. Immunol..

[16] Hamming O.J., Lutfalla G., Levraud J.-P., Hartmann R. (2011). Crystal structure of Zebrafish interferons I and II reveals conservation of type I interferon structure in vertebrates. J. Virol..

[17] Pereiro P., Costa M.M., Diaz-Rosales P., Dios S., Figueras A., Novoa B. (2014). The first characterization of two type I interferons in turbot (*Scophthalmus maximus*) reveals their differential role, expression pattern and gene induction. Dev. Comp. Immunol..

[18] Zou J., Gorgoglione B., Taylor N.G., Summathed T., Lee P.T., Panigrahi A., Genet C., Chen Y.M., Chen T.Y., Hassan M.U. (2014). Salmonids have an extraordinary complex type I IFN system: Characterization of the IFN locus in rainbow trout oncorhynchus mykiss reveals two novel IFN subgroups. J. Immunol..

[19] Sun B., Robertsen B., Wang Z., Liu B. (2009). Identification of an Atlantic salmon IFN multigene cluster encoding three IFN subtypes with very different expression properties. Dev. Comp. Immunol..

[20] Helfman G., Collette B., Facey D., Bowen B. (2010). The Diversity of Fishes.

[21] Aggad D., Mazel M., Boudinot P., Mogensen K.E., Hamming O.J., Hartmann R., Kotenko S., Herbomel P., Lutfalla G., Levraud J.P. (2009). The two groups of zebrafish virus-induced interferons signal via distinct receptors with specific and shared chains. J. Immunol..

[22] Casani D., Randelli E., Costantini S., Facchiano A.M., Zou J., Martin S., Secombes C.J., Scapigliati G., Buonocore F. (2009). Molecular characterisation and structural analysis of an interferon homologue in sea bass (*Dicentrarchus labrax* L.). Mol. Immunol..

[23] Maekawa S., Chiang Y.A., Hikima J., Sakai M., Lo C.F., Wang H.C., Aoki T. (2016). Expression and biological activity of two types of interferon genes in medaka (*Oryzias latipes*). Fish Shellfish Immunol..

[24] Long S., Milev-milovanovic I., Wilson M., Bengten E., Clem L.W., Miller N.W., Chinchar V.G. (2006). Identification and expression analysis of cDNAs encoding channel catfish type I interferons. Fish Shellfish Immunol..

[25] Stein C., Caccamo M., Laird G., Leptin M. (2007). Conservation and divergence of gene families encoding components of innate immune response systems in zebrafish. Genome Biol..

[26] Stewart W.E. (1980). Interferon nomenclature recommendations. J. Infect. Dis..

[27] Levraud J.P., Boudinot P., Colin I., Benmansour A., Peyrieras N., Herbomel P., Lutfalla G. (2007). Identification of the zebrafish IFN receptor: Implications for the origin of the vertebrate IFN system. J. Immunol..

[28] Sun B., Greiner-Tollersrud L., Koop B.F., Robertsen B. (2014). Atlantic salmon possesses two clusters of type I interferon receptor genes on different chromosomes, which allows for a larger repertoire of interferon receptors than in zebrafish and mammals. Dev. Comp. Immunol..

[29] Bergan V., Steinsvik S., Xu H., Kileng Ø., Robertsen B. (2006). Promoters of type I interferon genes from Atlantic salmon contain two main regulatory regions. FEBS J..

[30] Purcell M.K., Laing K.J., Woodson J.C., Thorgaard G.H., Hansen J.D. (2009). Characterization of the interferon genes in homozygous rainbow trout reveals two novel genes, alternate splicing and differential regulation of duplicated genes. Fish Shellfish Immunol..

[31] Chang M.-X., Zou J., Nie P., Huang B., Yu Z., Collet B., Secombes C.J. (2013). Intracellular interferons in fish: A unique means to combat viral infection. PLoS Pathog..

[32] Sang Y., Liu Q., Lee J., Ma W., McVey D.S., Blecha F. (2016). Expansion of amphibian intronless interferons revises the paradigm for interferon evolution and functional diversity. Sci. Rep..

[33] Boudinot P., Zou J., Ota T., Buonocore F., Scapigliati G., Canapa A., Cannon J., Litman G., Hansen J.D. (2014). A tetrapod-like repertoire of innate immune receptors and effectors for coelacanths. J. Exp. Zool. Part B Mol. Dev. Evolut..

[34] Palha N., Guivel-Benhassine F., Briolat V., Lutfalla G., Sourisseau M., Ellett F., Wang C.H., Lieschke G.J., Herbomel P., Schwartz O. (2013). Real-time whole-body visualization of Chikungunya Virus infection and host interferon response in zebrafish. PLoS Pathog..

[35] Feng H., Zhang Q.M., Zhang Y.B., Li Z., Zhang J., Xiong Y.W., Wu M., Gui J.F. (2016). Zebrafish IRF1, IRF3, and IRF7 Differentially Regulate IFNPhi1 and IFNPhi3 Expression through Assembly of Homo- or Heteroprotein Complexes. J. Immunol..

[36] Liao Z., Wan Q., Su J. (2016). Bioinformatics analysis of organizational and expressional characterizations of the IFNs, IRFs and CRFBs in grass carp *Ctenopharyngodon idella*. Dev. Comp. Immunol..

[37] Lopez-Munoz A., Roca F.J., Meseguer J., Mulero V. (2009). New insights into the evolution of IFNs: Activities genes and display powerful antiviral transient expression of IFN-dependent zebrafish group II IFNs induce a rapid and display powerful antiviral activities. J. Immunol..

[38] Svingerud T., Solstad T., Sun B., Nyrud M.L.J., Kileng Ø., Greiner-Tollersrud L., Robertsen B. (2012). Atlantic salmon type I IFN subtypes show differences in antiviral activity and cell-dependent expression: Evidence for high IFNb/IFNc-producing cells in fish lymphoid tissues. J. Immunol..

[39] Taniguchi T., Ogasawara K., Takaoka A., Tanaka N. (2001). IRF family of transcription factors as regulators of host defense. Annu. Rev. Immunol..

[40] Feng H., Zhang Y.B., Zhang Q.M., Li Z., Zhang Q.Y., Gui J.F. (2015). Zebrafish IRF1 regulates IFN antiviral response through binding to IFNvarphi1 and IFNvarphi3 promoters downstream of MyD88 signaling. J. Immunol..

[41] Sun F., Zhang Y.B., Liu T.K., Gan L., Yu F.F., Liu Y., Gui J.F. (2010). Characterization of fish IRF3 as an IFN-inducible protein reveals evolving regulation of IFN response in vertebrates. J. Immunol..

[42] Sun F., Zhang Y.B., Liu T.K., Shi J., Wang B., Gui J.F. (2011). Fish MITA Serves as a Mediator for Distinct Fish IFN Gene Activation Dependent on IRF3 or IRF7. J. Immunol..

[43] López-Muñoz A., Roca F.J., Sepulcre M.P., Meseguer J., Mulero V. (2010). Zebrafish larvae are unable to mount a protective antiviral response against waterborne infection by spring viremia of carp virus. Dev. Comp. Immunol..

[44] Boxx G.M., Cheng G. (2016). The Roles of Type I Interferon in Bacterial Infection. Cell Host Microbe.

[45] Van der Vaart M., van Soest J.J., Spaink H.P., Meijer A.H. (2013). Functional analysis of a zebrafish myd88 mutant identifies key transcriptional components of the innate immune system. Dis. Models Mech..

[46] Chang C.J., Sun B., Robertsen B. (2015). Adjuvant activity of fish type I interferon shown in a virus DNA vaccination model. Vaccine.

[47] Chang C.J., Robertsen C., Sun B., Robertsen B. (2014). Protection of Atlantic salmon against virus infection by intramuscular injection of IFNc expression plasmid. Vaccine.

[48] Ding Y., Ao J., Huang X., Chen X. (2016). Identification of Two Subgroups of Type I IFNs in Perciforme Fish Large Yellow Croaker Larimichthys crocea Provides Novel Insights into Function and Regulation of Fish Type I IFNs. Front. Immunol..

[49] Petit J.-L., Stange-Thomann N., Mauceli E., Bouneau L., Jaillon O., Aury J.-M., Ozouf-Costaz C., Bernot A., Nicaud S., Jaffe D. (2004). Genome duplication in the teleost fish Tetraodon nigroviridis reveals the early vertebrate proto-karyotype. Nature.

[50] Meyer A., Schartl M. (1999). Gene and genome duplications in vertebrates: The one-to-four (-to-eight in fish) rule and the evolution of novel gene functions. Curr. Opin. Cell Biol..

[51] Magadan S., Sunyer O.J., Boudinot P. (2015). Unique Features of Fish Immune Repertoires: Particularities of Adaptive Immunity Within the Largest Group of Vertebrates. Results Probl. Cell Differ..

[52] Trobridge G.D., Leong J.A. (1995). Characterization of a rainbow trout Mx gene. J. Interferon Cytokine Res..

[53] Hansen J.D., la Patra S. (2002). Induction of the rainbow trout MHC class I pathway during acute IHNV infection. Immunogenetics.

[54] Boudinot P., Massin P., Blanco M., Riffault S., Benmansour A. (1999). vig-1, a new fish gene induced by the rhabdovirus glycoprotein, has a virus-induced homologue in humans and shares conserved motifs with the MoaA family. J. Virol..

[55] Boudinot P., Salhi S., Blanco M., Benmansour A., Introduction I., America N. (2001). Viral haemorrhagic septicaemia virus induces vig-2, a new interferon-responsive gene in rainbow trout. Fish Shellfish Immunol..

[56] O’Farrell C., Vaghefi N., Cantonnet M., Buteau B., Boudinot P., Benmansour A. (2002). Survey of Transcript Expression in Rainbow Trout Leukocytes Reveals a Major Contribution of Interferon-Responsive Genes in the Early Response to a Rhabdovirus Infection. J. Virol..

[57] Briolat V., Jouneau L., Carvalho R., Palha N., Langevin C., Herbomel P., Schwartz O., Spaink H.P., Levraud J.P., Boudinot P. (2014). Contrasted innate responses to two viruses in zebrafish: Insights into the ancestral repertoire of vertebrate IFN-stimulated genes. J. Immunol..

[58] Ozato K., Shin D.M., Chang T.H., Morse H.C. (2008). TRIM family proteins and their emerging roles in innate immunity. Nat. Rev. Immunol..

[59] Yap M.W., Nisole S., Lynch C., Stoye J.P. (2004). Trim5alpha protein restricts both HIV-1 and murine leukemia virus. Proc. Natl. Acad. Sci. USA.

[60] Sawyer S.L., Emerman M., Malik H.S. (2007). Discordant Evolution of the Adjacent Antiretroviral Genes TRIM22 and TRIM5 in Mammals. PLoS Pathog..

[61] Mallery D.L., McEwan W.A., Bidgood S.R., Towers G.J., Johnson C.M., James L.C. (2010). Antibodies mediate intracellular immunity through tripartite motif-containing 21 (TRIM21). Proc. Natl. Acad. Sci. USA.

[62] Chelbi-Alix M.K., Vidy A., el Bougrini J., Blondel D. (2006). Rabies viral mechanisms to escape the IFN system: The viral protein P interferes with IRF-3, Stat1, and PML nuclear bodies. J. Interferon Cytokine Res..

[63] Boudinot P., van der Aa L.M., Jouneau L., Pasquier L.D., Pontarotti P., Briolat V., Benmansour A., Levraud J.P. (2011). Origin and evolution of TRIM proteins: New insights from the complete TRIM repertoire of zebrafish and pufferfish. PLoS ONE.

[64] Van der Aa L.M., Levraud J.-P., Yahmi M., Lauret E., Briolat V., Herbomel P., Benmansour A., Boudinot P. (2009). A large new subset of TRIM genes highly diversified by duplication and positive selection in teleost fish. BMC Biol..

[65] Sardiello M., Cairo S., Fontanella B., Ballabio A., Meroni G. (2008). Genomic analysis of the TRIM family reveals two groups of genes with distinct evolutionary properties. BMC Evolut. Biol..

[66] Kollmann T.R., Levy O., Montgomery R.R., Goriely S. (2012). Innate immune function by Toll-like receptors: Distinct responses in newborns and the elderly. Immunity.

[67] Castro R., Jouneau L., Tacchi L., Macqueen D.J., Alzaid A., Secombes C.J., Martin S.A., Boudinot P. (2015). Disparate developmental patterns of immune responses to bacterial and viral infections in fish. Sci. Rep..

